# Otology

**Published:** 1895-03-16

**Authors:** 


### OTOLOGY.

**External Muscles of the Ear.**—As a result of a study of the muscles of the external ear Ambrose Birmingham has found[1]: (1) That the tendinous band which lies on the superior curved line, and is said to represent the transversus nuchæ, gives origin to the middle portion of the occipitalis, and to the upper division of the retrahens auriculam. (2) That the occipitalis is made up of three parts—an internal, which arises from the curved line ; a middle, from the transversus nuchæ tendon ; and an outer, from the mastoid portion of the temporal bone ; and that the outermost fibres of the muscle are frequently connected to the ear, while the next fibres send their aponeurosis forward under the attollens. (3) That the retrahens, in its perfect condition, is composed of an upper division which arises from the mastoid in connection with the outer part of the occipitalis, and a lower which springs from the transversus nuchæ tendon. (4) That the attollens rises from the outer surface, not from the margin of the epicranial aponeurosis, and that it is inserted partly into the outer, partly into the deep surface of the pinna. (5) That the attrahens auriculam is a perfectly distinct and separate muscle, unconnected with the attollens ; that it lies beneath the temporal artery at a deeper level than that muscle, and that a connection can occasionally be traced between it and the upper part of the frontalis. (6) That a parotido-auricularis is present in a fair proportion of cases. (7) That the helicis major is an extrinsic muscle of the ear, connected above with the attollens. (8) That there is possibly a connection between the retrahens on the one hand and the obliquus and transversus on the other. (9) That there is a deep fascia in the scalp between the epicranial aponeurosis and the superficial fascia.

**A bacteriological examination of the purulent ear discharge** occurring in scarlet fever has been undertaken[2] by F. R. Blaxall. Out of the fourteen cases chosen for examination, the various organisms were found as follows: Streptococcus pyogenes, twelve times; bacillus striatus albus, nine times; staphylococcus pyogenes albus, eight times; staphylococcus pyogenes aureus, five times; micrococcus albus liquefaciens, three times; bacillus acidi lactici, twice; bacillus subtilis, once; bacillus pyocyaneus, once; bacillus tuberculosis, once; yeasts, six times; sarcinæ, three times; moulds, four times; and a few other bacteria whose recognition was not absolute, and which were found to be non-pathogenic for mice. It appears from his observations: (1) That the organism most potent in the etiology of the otitis media of scarlet fever is the streptococcus pyogenes. (2) That the less chance there is of contamination from the outer air through the external orifice the more the pyogenic cocci predominate over rod forms, but that prior to perforation of the membrane the occurrence of such organisms is not precluded, since they may ascend from the mouth and air passages. (3) That next to the streptococcus the most important organisms are the staphylococci albus and aureus. (4) That apparently the diplococcus pneumoniæ of Fraenkel or the bacillus pneumoniæ of Friedlander do not play such an important part in the otitis media of scarlet fever as in that due to other causes.

**Acoustic Exercises** in the treatment of deaf-mutism have been practised[3] by Professor Urbantschitsch. A beginning is made by pronouncing in the ear of the patient two vowels in a loud voice—"a" and "i," for instance; and these vowels are repeated until the child is able to distinguish both sounds, after which other vowels are treated in the same way, then consonants, words, and finally whole sentences. An exercise of from five to ten minutes three or four times a week is sufficient to greatly improve hearing. Since October, 1893, Urbantschitsch has employed this method in 60 children. Of these none could distinguish sentences, 6 perceived words, 22 vowels, and 32 had only traces of audition left. At the present time 12 of the children perceive sentences, 16 words, 2 vowels, and 11 have only traces of audition. A somewhat similar method has been adopted[4] in 30 cases by Coen. He does not employ it on those who are completely deaf and dumb, but only on those who have suffered from more or less severe deafness and consecutive total or partial inability to speak, or from general stammering, and who have come under his treatment on account of their defect of speech. Allied to these means is the aural massage of Bissell,[5] who uses a telephone receiver attached to a Gotel battery, which gives a large range of vibrations by means of a ribbon rheotome, which can vary from 60 to 20,000 per minute.

**Cholesteatoma of the Ear.**—Friedenwald, of Baltimore considers[6] it important to remember that there is a tendency for cholesteatoma or cholesteatomatous, masses to recur. Patients are therefore to be examined at intervals for some time after apparent cure. The treatment consists in their thorough removal, with complete antiseptic cleansing of the drum cavity by the ordinary well-known means.

**Abscess of Fixation.**—H. Colladon, in presenting[7] a paper on this subject, gives credit to Szenes, of Buda-Pesth, as having been the first to draw attention to a secondary diffuse external otitis occurring as a complication in a certain percentage of cases of acute suppurative otitis media, and to its probable curative action on the otorrhœa.[8] The author's conclusions may be summed up as follows[9]: (1) Abscess of fixation is a proceeding of nature by which inflammation with a tendency to suppuration of an organ deeply seated is derived to the periphery, and from this the cure of the primary affection follows. (2) The auditory organ participates in the benefits of this process. (3) Acute median suppurative otitis may be terminated spontaneously by the formation of a diffuse external otitis, the cure of which at the end of three to four days is followed immediately by the cessation of the otorrhœa and the cicatrization of the perforation. (4) External otitis "of fixation" may be artificially induced by the injection and instillation into the ear of drugs, amongst which the irritant antiseptics, and especially thymic acid, appear to occupy the first rank. (5) Medication by "fixation" is efficacious beyond everything in acute median suppurative otitis. No otorrhœa should be allowed to last unchanged more than two or three weeks before trying this method. It deserves to be made the subject of experiment, however, in chronic suppurative median otitis free from complications.

**Otitis Media of Infants** has been investigated by Hartmann and Kossel,[10] who have found that: (1) Post-mortem examination and examination of the ears of living children establish the fact that 75 per cent. suffer from inflammation of the middle ear. (2) Inflammation of the middle ear can nearly always be detected by an otoscopic examination. (3) The symptoms consist in restlessness, elevated temperature, and loss of weight, but sometimes these symptoms are absent. (4) Very often the symptoms are connected with broncho-pneumonic processes, which probably are both due to the same cause, viz., aspiration. (5) Death can result, in cases of otitis media, from slow atrophy or from an extension of the micro-organisms into the cranial cavity (meningitis) or into the blood (septicæmia). (6) The treatment must vary according to the conditions present.

Rasch[11] has also investigated this subject, having made sixty-one autopsies of children under two years of age. In but five cases was the tympanum normal, suppurative disease being present on one or both sides in forty-six, equalling 75 per cent.; and catarrhal lesions in eight cases, or 14 per cent. Nearly all of the children who had died of bronchopneumonia showed otitis, and in thirty-three of the forty-three in which the secretion was rigidly examined the pneumococcus was present. Not a few of these children had shown symptoms leading to a diagnosis of meningitis, which was found absent on section, and the tympanic inflammation had been generally overlooked. Perforation of the drum head was found but four times, and even when this occurred, the condition was too frequently unrecognised or slighted. This was the more deplorable when measures as simple as hot douching of the ear would often suffice to cure the disease in its early controllable stage. Woakes[12] in treating on this subject considers that implication of the ear organs in the exanthemata is one of the most frequent causes of fatal issue in these cases.

**Non-Suppurative Otitis Media.**—In opening a discussion[13] on the prognosis of this affection when associated with imperforate membrane, G. P. Field summed up his conclusions as follows[14]: (1) Prognosis in chronic middle ear catarrh is most favourable in children and young adults, in whom the cause is plainly attributable to local and removeable obstruction, nasopharyngeal or faucial abnormalities, or to simple mucous obstruction of the Eustachian tube from a common cold, or other temporary catarrhal condition, the result of inflation and other tests, satisfying us that secondary changes have not yet occurred to impede the function of the membrane and ossicles. (2) When, from whatever cause arising, or however long or short the duration, or from the age of the patient and other circumstances, the hearing power after inflation is recovered in part only, the inference is that consecutive changes due to organisation of secretions have already commenced, and that, slowly or quickly, the disease will continue to develop, no matter what treatment be adopted. (Roosa thinks that about 20 per cent. adult patients are relieved, but none absolutely cured.) (3) The prognosis is unfavourable where, with much deafness, there is no improvement whatever after forcible catheterisation, dilatation of the Eustachian tubes, removal of secretions or the intratympanic injection of solvents. (4) The prognosis is absolutely bad (still as regards improvement) when the symptoms point to primary sclerosis; and worst of all (as regards in their case retention of any hearing power) where, with or without sclerosis, the tuning-fork tests point to serious labyrinthine disturbance. In the *treatment* of non-suppurative disease of the middle ear Dr. Lautenbach strongly urges the use of massage as opposed to removal of the sound-conducting apparatus. He claims[15] that: (1) The method is not difficult to apply. (2) It is free from risk. (3) It has improved the hearing in over ninety per cent. of the cases. (4) In about ninety per cent. the tinnitus has been relieved. (5) It has removed the vertiginous symptoms in a little over half the cases.

**Chronic Tympanic Vertigo** is treated by Burnett[16] by removal of the incus, by which the retractive power of the tensor tympani muscle, and the malleus acting through the incus upon the stapes, is abrogated. The stapes is thus liberated, and the stapedius muscle, relieved now of the antagonism of the tensor tympani, acts more freely in drawing the stapes from the oval window, and retaining it in a normal position. Thirteen cases have thus been operated upon by Burnett, with most encouraging results, especially in the worst cases, viz., those in which the subjects had been suddenly attacked with vertigo. More or less relief has accrued in all cases, without any intercurrent and undesirable symptoms. In seven other cases of tympanotomy and removal of incus he has found, at the time of operation, mobility of the stapes, notwithstanding the usual symptoms of chronic catarrh of the middle ear. Tinnitus and vertigo have been relieved in all of these, but the deafness has remained unchanged, notwithstanding the persistent mobility of the stirrup.

**Meniere's Disease** has been treated by Labit[17] by hypodermic injections of pilocarpin. He reports three cases. In all there were the typical symptoms, noises, vertigo, nausea or vomiting, and deafness to osseal as well as aërial sound vibrations. The first case received fifteen injections of from $\frac{1}{26}$ to $\frac{1}{6}$ of a grain, the second thirteen of from $\frac{1}{13}$ to $\frac{1}{3}$ of a grain, and the third eight of from $\frac{1}{26}$ to $\frac{1}{4}$ of a grain continued and increased. In all the cases the vertigo disappeared, the noises diminished, and the hearing was, to a certain extent, restored. The writer compares the absorption produced to that observed in pleural and peritoneal effusions under the action of pilocarpin. Success depends upon the correctness of the diagnosis and the early adoption of the treatment. Baron[18] has also treated four cases of labyrinthine disease by this method, and reports favourably thereon.

[1] Dublin Journ. Med. Sci., Oct., 1894. [2] Brit. Med. Journ., July 21st, 1894, p. 116. [3] Med. Weekly, May 4th, 1894, p. 214. [4] Wien. Med. Woch., No. 5, 1894, and Brit. Med. Journ., May 19th, 1894. [5] Journ. of Laryngol., June, 1894, p. 376. [6] Med. News, Vol. 62, No. 10, and Am. Journ. Med. Sci., June, 1894, p. 727. [7] Revue. Med. de la Suisse Romande, Oct. 20th, 1894. [8] Brit. Med. Journ., Dec. 1st, 1894. [9] Journ. of Laryngol., Rhin. and Otology, May, 1894. [10] Deutsch. Med. Woch., 1894, No. 26, and Am. Journ. Med. Sci., Dec., 1894, p. 726. [11] Jahrbuch fur Kind., Vol. 37, Nos. 3 and 4, 1894, and Therapeut. Gazette, May 15th, 1894, p. 348. [12] Clinical Journ., Nov. 21, 1894, p. 59. [13] Annual Meeting of the Brit. Med. Assoc., held at Bristol, 1894. [14] Brit. Med. Journ., Nov. 24th, 1894, p. 1,157. [15] N. Y. Med. Journ., Nov. 10th, 1894. [16] Internat. Med. Mag., June, 1894, p. 343. [17] Brit. Med. Journ., Sep. 29th, 1894. [18] Brit. Med, Journ., Dec. 1st, 1894.

